# Consistent decrease in conifer embolism resistance from the stem apex to base resulting from axial trends in tracheid and pit traits

**DOI:** 10.3389/fpls.2024.1414448

**Published:** 2024-06-25

**Authors:** Dario Zambonini, Tadeja Savi, Sabine Rosner, Giai Petit

**Affiliations:** ^1^ Dept. Territorio e Sistemi Agro-Forestali, Università degli Studi di Padova, Legnaro (PD), Italy; ^2^ Department of Integrative Biology and Biodiversity Research, University of Natural Resources and Life Sciences, Vienna (BOKU), Institute of Botany, Vienna, Austria

**Keywords:** embolism, cavitation, xylem, vulnerability curve, P50, tracheids, lumen diameter, pits

## Abstract

**Introduction:**

Drought-induced embolism formation in conifers is associated with several tracheid and pit traits, which vary in parallel from stem apex to base. We tested whether this axial anatomical variability is associated with a progressive variation in embolism vulnerability along the stem from apex to base.

**Methods:**

We assessed the tracheid hydraulic diameter (*Dh*), mean pit membrane area (*PMA*) and the xylem pressure at 50% loss of conductivity (*P50*) on longitudinal stem segments extracted at different distances from the stem apex (*DFA*) in a *Picea abies* and an *Abies alba* tree.

**Results:**

In both trees, *Dh* and *PMA* scaled with *DFA* ^0.2^. *P50* varied for more than 3 MPa from the treetop to the stem base, according to a scaling of -*P50* with *DFA^-0.2^*. The largest *Dh*, *PMA* and *P50* variation occurred for *DFA*<1.5 m. *PMA* and *Dh* scaled more than isometrically (exponent *b*=1.2). Pit traits vary proportionally with tracheid lumen diameter.

**Discussion and conclusions:**

Apex-to-base trends in tracheid and pit traits, along with variations in P50, suggest a strong structure-function relationship that is influenced by *DFA*. Although the effect of *DFA* on *P50* has not been extensively explored previously, we propose that analyzing the relationship between *P50* and *DFA* could be crucial for a comprehensive assessment of embolism vulnerability at the individual level.

## Introduction

Increasing drought-related tree mortality is a global ecological crisis that demands urgent action from the scientific community. Trees most commonly die from xylem dysfunction by gas-embolism, causing strong limitations to leaf water supply, transpiration, and photosynthesis (Adams et al., 2017; Choat et al., 2018; Savi et al., 2019; Hammond, 2020). Interdisciplinary efforts are necessary to develop risk assessments that identify species-specific vulnerability to drought, enabling a better prediction of climate change impacts on forest ecosystems and global biogeochemical cycles.

### The mechanism of drought-induced embolism formation

Water moves from roots to leaves following gradients of sub-atmospheric pressure and flows through adjacent vascular conduits. Cohesion and adhesion forces between water molecules and xylem conduit walls maintain the liquid phase of water under tension (Tyree and Zimmermann, 2002). Xylem sap contains tiny gas bubbles (Schenk et al., 2016) that can cause embolization of the xylem conduit when exceeding a critical bubble diameter (Cochard, 2006). Bordered pits, which connect adjacent vascular elements (vessels, tracheids, and fibers in angiosperms; only tracheids in gymnosperms), serve as a barrier to prevent the entry of large air bubbles into functional elements. The anatomical properties of these pits play a crucial role in determining the vulnerability of xylem to drought-induced embolism formation (Bouche et al., 2014; Lens et al., 2022).

Bordered pits in angiosperms have simple homogeneous membranes with tiny pore constrictions, while pits of most conifers have a larger membrane structure with an outer permeable area (margo) and an inner, impermeable disc (torus) larger than the pit aperture. In conifers, when a gas bubble expands and embolizes a tracheid, the pit membranes get aspirated towards the functional conduit due to the pressure difference between the embolized and the functional tracheid (Tyree and Zimmermann, 2002). Consensus among plant physiologists indicates that drought induced embolization in conifer tracheids predominantly occurs through air-seeding. This process involves air from an already embolized tracheid protruding into an adjacent functional tracheid, forcing the displacement of the torus due to excessive tension (Delzon et al., 2010). The margo flexibility and the ratio of torus to pit aperture diameter (i.e., the torus overlap) contribute to a valve effect that seals the pit aperture of the functional tracheid when neighboring tracheids become embolized (Delzon et al., 2010; Bouche et al., 2014).

The estimation of embolism vulnerability is commonly done by measuring the percent loss of xylem hydraulic conductivity (*PLC*) at a specific xylem tension. The contribution of pits to the overall conduit conductance depends on various factors, including the number of pits, their size, and their membrane permeability. Previous studies have suggested that lumen and end-wall resistances contribute approximately 40% and 60%, respectively, to the total xylem resistance, irrespective of conduit size, in both angiosperms and gymnosperms (Sperry et al., 2006). This finding implies the existence of consistent relationships between lumen diameter and pit anatomical traits (Becker et al., 2003; Schulte, 2012; Lazzarin et al., 2016). However, it is important to note that similar levels of extra-lumen resistance can be achieved through different combinations of traits. In angiosperms, resistance is primarily influenced by the type and number of perforation plates per vessel, as well as the density, area, and permeability of pit membranes, and the presence of vestures. In conifers, resistance is determined by the density and area of pits, the permeability and area of the margo, and the size of the aperture area.

The resistance against drought-induced embolism formation is primarily determined by key pit traits (Delzon et al., 2010; Bouche et al., 2014), leading to a trade-off between hydraulic efficiency and safety (Pittermann et al., 2006). Although this trade-off should be expected to occur mainly at the individual or intra-specific level, it has often been investigated at the interspecific level. One reason why global analyses have failed to provide robust empirical support for the hydraulic efficiency-safety trade-off is the oversight of species-specific combinations of lumen and pit traits (Gleason et al., 2016). Additionally, these types of meta-analyses involve the association of hydraulic and anatomical data obtained using different methods and sampling approaches, such as sampling at fixed branch age or diameter.

These approaches, which are still most commonly used by plant physiologists, do not consider the patterns of variation in anatomical traits that have recently been clearly demonstrated (see the next section). In the last decade, significant progress has been made to advancing technical capabilities and improving measurement protocols for assessing xylem vulnerability to drought-induced embolism (Cochard et al., 2013; Johnson et al., 2022). However, the contemporary advances in understanding the variability of different xylem anatomical traits and their covariation at individual, intra-specific, and interspecific levels have not received the deserved attention, even though some studies already provided evidence that the xylem vulnerability to drought-induced embolism formation strongly increases axially along the stem from the treetop to the base (Domec and Gartner, 2001).

### The xylem anatomical traits vary axially from the stem apex to base

It has been widely demonstrated that leaves and roots are hydraulically connected through the xylem transport system, with vascular conduits being narrowest at the distal end of the hydraulic path and progressively widening basally along the leaf venation network (Lechthaler et al., 2020) and further down along the outermost, youngest, and longest xylem layer in the stem (Anfodillo et al., 2013). Belowground, vascular conduits along the roots are generally wider than those in the stem (McElrone et al., 2004; Petit et al., 2009, 2010; Jacobsen et al., 2018).Unlike stem conduits, the pattern of axial widening in roots is highly variable (Prendin et al., 2018). The limited research on this topic has not suggested a clear convergence toward a general pattern.

In the stem, the pattern of widening is consistently repeated every year of growth (Petit et al., 2023) and also occurs radially from the innermost to the outer xylem layers due to continuous height growth during ontogeny (Carrer et al., 2015). Notably, the axial distance from the distal apex (*DFA*) is the best predictor of conduit diameter, according to a power scaling relationship (*Y=a×X^b^*) with an exponent that is nearly invariant at the individual level (Prendin et al., 2018; Petit et al., 2023) and varies only slightly across species (Anfodillo et al., 2013; Koçillari et al., 2021).

Moreover, studies in conifers have reported significant relationships between tracheid lumen diameter and other anatomical features, such as cell wall thickness (Sperry et al., 2006; Bouche et al., 2014; Prendin et al., 2018), tracheid length and number, chamber aperture, torus areas of pits (Becker et al., 2003; Sperry et al., 2006; Lazzarin et al., 2016; Held et al., 2021), torus overlap, and margo flexibility (Delzon et al., 2010; Song et al., 2022). Coherently, a few studies showed that pit traits vary axially along the stem seemingly to conduit diameter (Schulte, 2012; Lazzarin et al., 2016).

Despite the importance of these allometric relationships for xylem hydraulic efficiency and embolism resistance, they have seldom been investigated and rarely considered in physiological studies.

### The limited knowledge on axial variation in xylem hydraulic traits

Although xylem hydraulic traits are commonly investigated on small plants or at the level of distal branches in mature trees, yet it remains not fully investigated whether these trait variations at the individual level can be explained by known patterns of xylem anatomical traits, such as the scaling of conduit diameter or pit properties with *DFA*. The limited investigations on mature trees have shown apparently contradictive results. Some studies reported embolism vulnerability to be lower in terminal twigs than in the stem bole (Domec and Gartner, 2001; Spicer and Gartner, 2001; Domec et al., 2009b; Rosner et al., 2019b). An analysis conducted on *Pseudotsuga menziesii* (Mirb.) Franco reported that the water potential corresponding to 50% loss of xylem hydraulic conductivity (*P50*) decreased from -3.3 MPa at the stem base to -4.7 MPa at the level of the fifth internode starting from the stem apex (Domec and Gartner, 2001). Furthermore, *P50* was observed to decrease with sapwood depth (Spicer and Gartner, 2001) or being higher at the base of tall trees compared to short trees (Spicer and Gartner, 2001; Olson et al., 2018). In parallel, xylem specific conductivity (*ks*) was reported to increase basally along the stem from the apex downwards and from inner to outer sapwood (Spicer and Gartner, 2001; Domec et al., 2012). On the contrary, a recent investigation found that narrow branches exhibit lower vulnerability compared to the stem, which showed no variation in vulnerability across longitudinal segments of the outermost sapwood extracted at different heights along the main trunk of mature trees (Bouche et al., 2016). Furthermore, in a few temperate broadleaved species the xylem specific hydraulic conductivity was reported to be lower in apical branches than in roots, consistent with anatomical differences, whereas the embolism vulnerability resulted similar between these organs (Lübbe et al., 2022).

Instead, other studies reported individual variability in embolism vulnerability in small plants (of ~ 1 m height) that did not align with commonly observed trends in anatomical traits. For instance, the estimated *P50* in saplings was noted to be rather comparable between stems and roots in certain broadleaved species (Creek et al., 2018; Rodriguez-Dominguez et al., 2018), or even lower (i.e., more negative) in stems compared to roots, despite the former possessing smaller vessels with smaller pits (Wu et al., 2020).

Notably, the hydraulic techniques used varied not just between the different studies, but sometimes even within a single study.

This study aims to provide empirical evidence for how *DFA* affects the variability of anatomical (conduit diameter and pit area) and hydraulic traits (*P50*) at the individual level in conifer trees. We discuss the importance of using an allometric approach in studies of xylem hydraulics to account for possible axial patterns in anatomical and hydraulic traits, and thus use the scaling parameters to more properly characterize the anatomical and hydraulic traits at the individual and species level.

## Materials and methods

### Sampling

For this study, two mature conifers were selected: a 30-meter silver fir (*Abies alba* Mill.) from a mixed conifer forest located in Asiago, Italy (45° 54’ 6” N, 11° 29’ 22” E, 1320 m a.s.l.) and a 25-meter Norway spruce (*Picea abies* Karst.) from a pure spruce forest in Enicklberg, Niederösterreich, Austria (48° 14’ 36” N, 15° 28’ 40” E, 550 m a.s.l.). Both trees were felled in May 2022. Stem discs (19 in the *A. alba* and 25 in the *P. abies* tree) of ~ 25 cm in length were immediately extracted from at different heights along the stem, and their distance from the stem apex (*DFA*) determined. Due to the limited number of samples obtainable within the first 1.5 meters from the apex, we measured additional segments along the main axis of side branches within 1.5 m from the branch apex. These samples were obtained from branches of the second or third node to minimize possible effects of shade and bending on wood anatomical traits (in those cases *DFA* referred to the distance from the branch apex). Samples were first debarked, and then sealed in plastic bags with wet paper and transported to the laboratory. For each stem disc, 1–5 sticks of 3 × 5 ×15 cm containing the outermost sapwood rings were obtained from intact and healthy regions with no reaction wood. Apical segments with a diameter< 1 cm were not further processed. All segments were enclosed in vacuum-sealed bags and stored at -20°C. The day before measurements, segments were soaked in distilled water for 24 hours under vacuum. After this rehydration process, samples were split along the fibers to obtain sticks of approximately 0.8 × 0.8 × 14 cm. These sticks contained the three outermost sapwood rings in most cases. The surfaces of the sticks were smoothed with microtome blades. Finally, the sticks were soaked in filtered distilled water purified with silver ions (Micropure, Katadyn Products, Wallisellen, Switzerland) under low-vacuum at room temperature overnight to rehydrate the tissue and refill previously embolized (i.e., air-filled) tracheids (Rosner et al., 2018, 2019a). Previous analyses on Norway spruce sapwood samples have preliminarily tested and found that freeze-storage of saturated samples had no significant impact on specific hydraulic conductivity and vulnerability after thawing (Rosner et al., 2006, but these results were not shown). Additionally, our own unpublished data from a follow-up study on the axial variability in embolism vulnerability in *Acer pseudoplatanus* L. also found no discernible effects of freeze-storage on vulnerability curves.

### Hydraulic measurements

Vulnerability curves (*VCs*) were assessed by using the air injection technique (Rosner et al., 2021; Savi, 2023). This technique has been commonly used for measuring hydraulic vulnerability of incised sapwood samples from conifer trunks (Domec and Gartner, 2001; Domec et al., 2009a; Rosner et al., 2019b). Sticks were re-cut under water at both ends with sharp razor blades, and connected to a reservoir (at 0.8 m height) containing distilled water with silver ions. The maximum sample hydraulic conductance (*Kmax*) was measured gravimetrically at 8 kPa by collecting sap at the distal end using pre-weighed vials containing a piece of sponge (five vials, 30 seconds interval). Next, the sticks were inserted into a double-ended pressure sleeve (PMS Instruments, Corvallis, OR, USA) and subjected to a pressure of 0.2–0.5 MPa for one minute. The sticks were thus allowed to equilibrate in water for 20 minutes, and the hydraulic conductivity (*K_i_*) was measured again as described above. This process was repeated at increasing pressures, and the percent loss of conductance (*PLC_i_*) was then calculated as:


(1) $$PLC_{i}=\left(1-\frac{K_{i}}{K_{max}}\right)\times 100$$

where *K_i_* is the sample conductance measured after the *i*-step of pressurization. The pressure applied in subsequent air injection cycles was gradually increased by steps of 0.5 - 1.0 MPa until *PLC_i_* > 90% was reached.

### Anatomical measurements

Anatomical subsamples of 2 cm were obtained from the center of each stick previously used for hydraulic measurements. These subsamples were cut on cross-section for tracheid lumen diameter measurements and on the longitudinal-radial section for pit measurements using a rotary microtome (LEICA RM2245, Leica Biosystems, Nusslock, Germany) at 10–12 μm, stained with safranine and AstraBlue, and permanently fixed with Eukitt (BiOptica, Milan, Italy). Slides were scanned at 20x with a slide scanner (Axioscan7, Carl Zeiss Microscopy GmbH, Germany). Cross-sections were analyzed with ROXAS (von Arx and Carrer, 2014), which automatically measure the hydraulically weighted mean conduit diameter (*Dh*=*Σd_i_^5^*/*Σd_i_^4^*, where *d_i_* is the diameter of the *i*-conduit, Kolb and Sperry, 1999).

To determine the average surface area of single inter-tracheid pit membranes (*PMA*), radial sections were analyzed using ImageJ software (National Institutes of Health, Bethesda, MD, USA). Specifically, the contour of the pit membrane area was manually drawn on 30–50 pits per sample.

### Statistical analysis and assessment of tracheid vulnerability curve

*VCs* were assessed by fitting *PLC* vs. *P* data by using the fitplc package (Duursma and Choat, 2017) in the R software (R Development Core Team, 2022). For each *VC*, the *P50* value (corresponding to the applied pressure at which *PLC* = 50%) was extracted.

The allometric relationships between the measured traits were analyzed by using power scaling relationships:


(2) $$Y=a\times X^{b}$$

where *a* is the allometric constant and *b* the scaling exponent. Data were first log_10_-transformed to comply with the assumption of normality and homoscedasticity (Zar, 1999), and then fitted with linear regressions, as commonly done in allometric analyses. Equation 2 was then linearized as:


(3) $$\log_{10}Y=\log_{10}a+b\times \log_{10}X$$

In this way, the allometric constant (*a*) and exponent (*b*) of Equation 2 become the *y*-intercept (=*log_10_a*) and slope (*b*) of Equation 3, respectively. The effects of *log_10_DFA*, tree ID and their interaction on hydraulic (*log_10_P50*) and anatomical traits (*log_10_Dh*, *log_10_PMA*) were tested by using linear mixed effects models fitted with restricted maximum likelihood (REML) by using the lme4 R-package (Bates et al., 2015). The best model was chosen based on the Akaike Information Criterion (AIC) (Zuur et al., 2009). Sample ID was used as random factor in all models.

The list of variables used in the hydraulic and anatomical analyses is reported in **Table 1**.

**Table 1:** List of variables.

Acronym	Unit	Description
*DFA*	cm	Axial distance from the stem (or branch) apex
Anatomical variables		
*Dh*	µm	Hydraulically weighted mean tracheid diameter (*Dh=Σd^5^/Σd^4^*)
*PMA*	µm^2^	Average circular area of tracheid pits
Hydraulic variables		
*PLC*	%	Percent loss of sample conductance
*P50*	MPa	Xylem pressure at which PLC=50%

## Results

**Figure 1:**
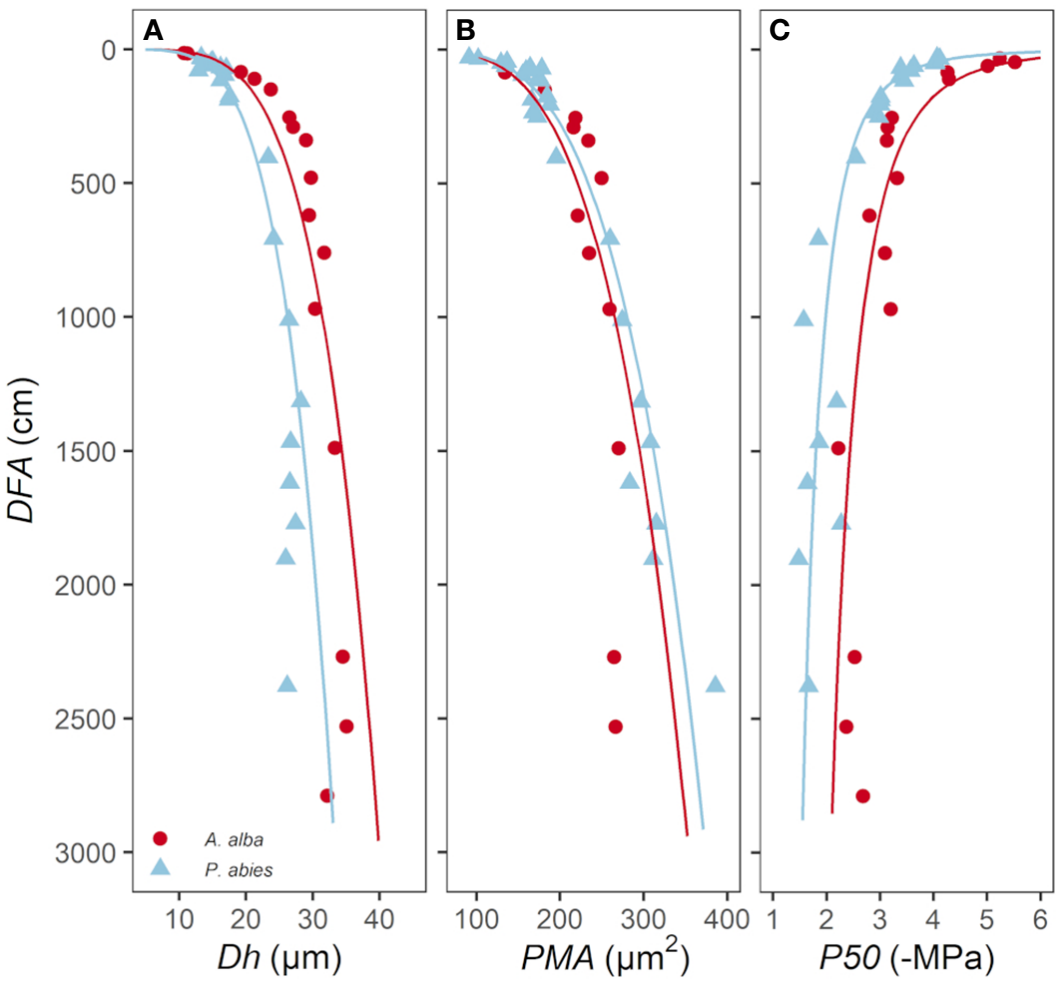
Variation in **(A)** *Dh*, **(B)** *PMA* and **(C)** *P50* with *DFA* in *A*. *alba* (blue triangles) and *P. abies* (red circles). Fitting lines are according to **Table 2**.

The hydraulically weighted diameter (*Dh*) and the average area of the intertracheid pit membranes (*PMA*) and the water potential inducing the 50% of conductivity loss (*P50*) showed a large variability down along the stem from apex to base (**Figure 1**).

**Figure 2:**
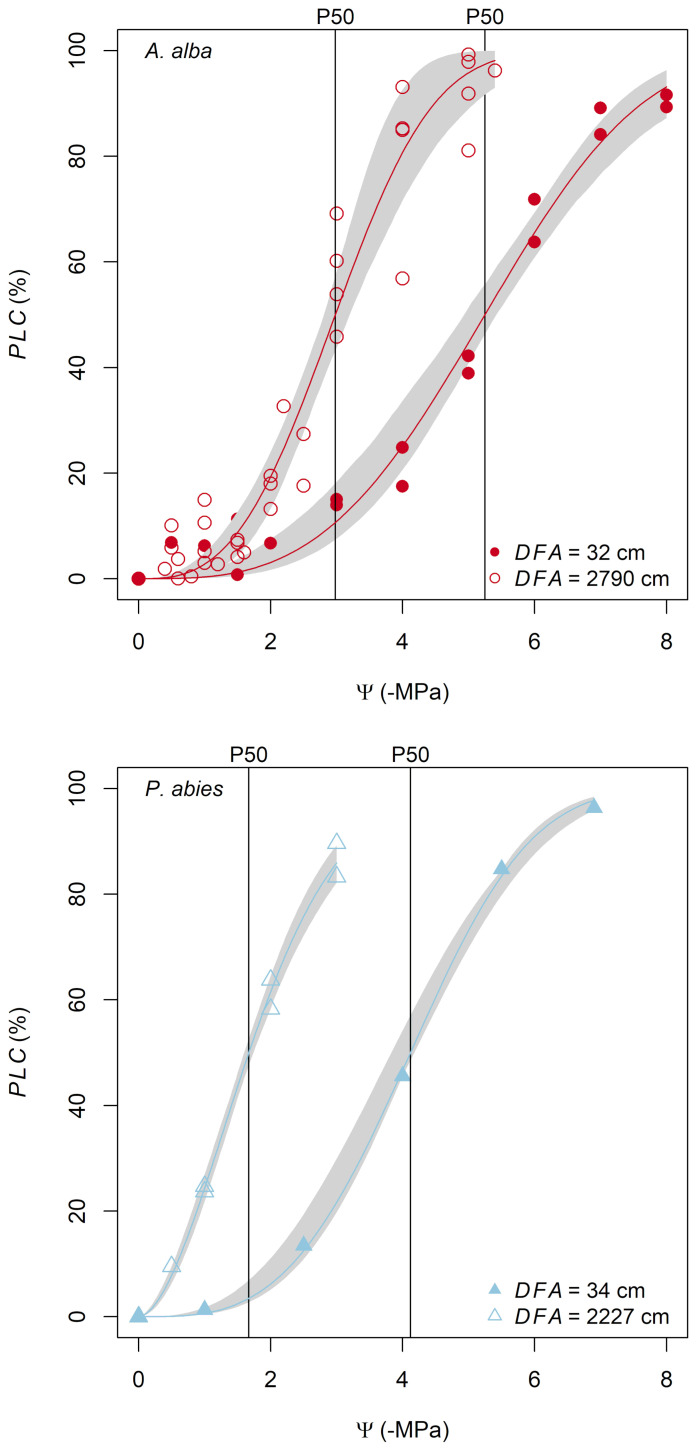
Hydraulic vulnerability curves obtained from the apical and basal stem samples (the distance from the apex, *DFA*, is reported in the legend) for both *A. alba* and *P. abies* trees.

*P50* values ranged from -4.0 to -1.5 MPa for the *P. abies* tree and from -5.5 to -2.2 MPa for the *A. alba* tree (**Figure 2**).

**Figure 3:**
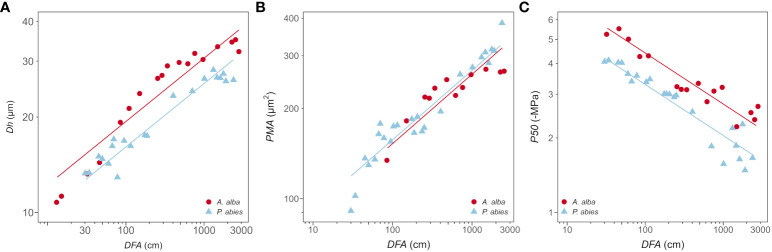
Variation in **(A)** *Dh*, **(B)** *PMA* and **(C)** *P50* with *DFA* in *A*. *alba* (blue triangles) and *P. abies* (red circles). Fitting lines are according to **Table 2**. *x* and *y* axis are shown with log_10_ scale.

The anatomical traits *Dh* and *PMA* increased with *DFA* according to power relationships (*Dh*~*DFA* ^0.19^ and *PMA*~*DFA* ^0.23^), whose exponent *b* (Equation 2) was similar in both species (**Figures 3A, B**; **Tables 2A, B**). According to our statistical model, the effects of *DFA* and tree *ID* accounted for 92% of the total *Dh* variance and 88% of the total *PMA* variance. The model accurately described the axial variation of these traits from the stem apex to ~800 cm down the stem, but it slightly overestimated them below this point to the base.

**Table 2:** Results of the linear mixed-effects models with best AIC predicting the effects of *log_10_DFA* and *Tree ID* on (A) *log_10_Dh*, (B) *log_10_PMA* and (C) *log_10_P50*.

A	Model: *log_10_Dh ~ log_10_DFA + Tree ID + (Sample ID)*					
	fixed effects	Value	Std. error	DF	t value	p value
	*y*-intercept	0.89	0.03	36	33.36	<0.0001
	*log_10_DFA*	0.20	0.01	36	19.75	<0.0001
	*Tree ID (P. abies)*	-0.08	0.01	36	-5.74	<0.0001
	*R^2^marginal* = 0.92 *R^2^conditional* = 0.99					
**B**	Model: *log_10_PMA ~ log_10_DFA + Tree ID + (Sample ID)*					
	Fixed effects	Value	Std. error	DF	t value	p value
	*y*-intercept	1.72	0.04	34	40.96	<0.0001
	*log_10_DFA*	0.23	0.01	34	16.01	<0.0001
	*Tree ID (P. abies)*	0.01	0.02	34	0.71	0.4796
	*R^2^marginal* = 0.88 *R^2^conditional* = 0.99					
**C**	Model: *log_10_P50 ~ log_10_DFA + Tree ID + (Sample ID)*					
	Fixed effects	Value	Std. error	DF	t value	p value
	*y*-intercept	1.06	0.03	36	32.58	<0.0001
	*log_10_DFA*	-0.21	0.01	36	-17.41	<0.0001
	*Tree ID (P. abies)*	-0.13	0.01	36	-8.51	<0.0001
	*R^2^marginal* = 0.90 *R^2^conditional* = 0.99					

*Sample ID* was used as random factor.

*Dh* proportionally increased from apex to base at similar rates in both trees (i.e., same exponent *b*=0.23), but at any *DFA* position in the stem it was significantly narrower in our *P. abies* tree than the *A. alba* tree (i.e., lower *y*-intercept) (**Figure 3A**; **Table 2A**).

Instead, there were no significant differences in the axial pattern of *PMA* along the stem between our sampled trees. *PMA* proportionally increased from apex to base at similar rates in both *P. abies* and *A. alba* trees (i.e., same exponent *b*=0.23). The model predicted that at any *DFA* position in the stem *PMA* was significantly similar between both trees (i.e., similar *y-*intercept). However, in our *P. abies* tree, *PMA* did not to vary for *DFA* > 800 *cm*, appearing smaller than in *A. alba* towards the stem base (**Figure 3B**; **Table 2B**).

**Figure 4:**
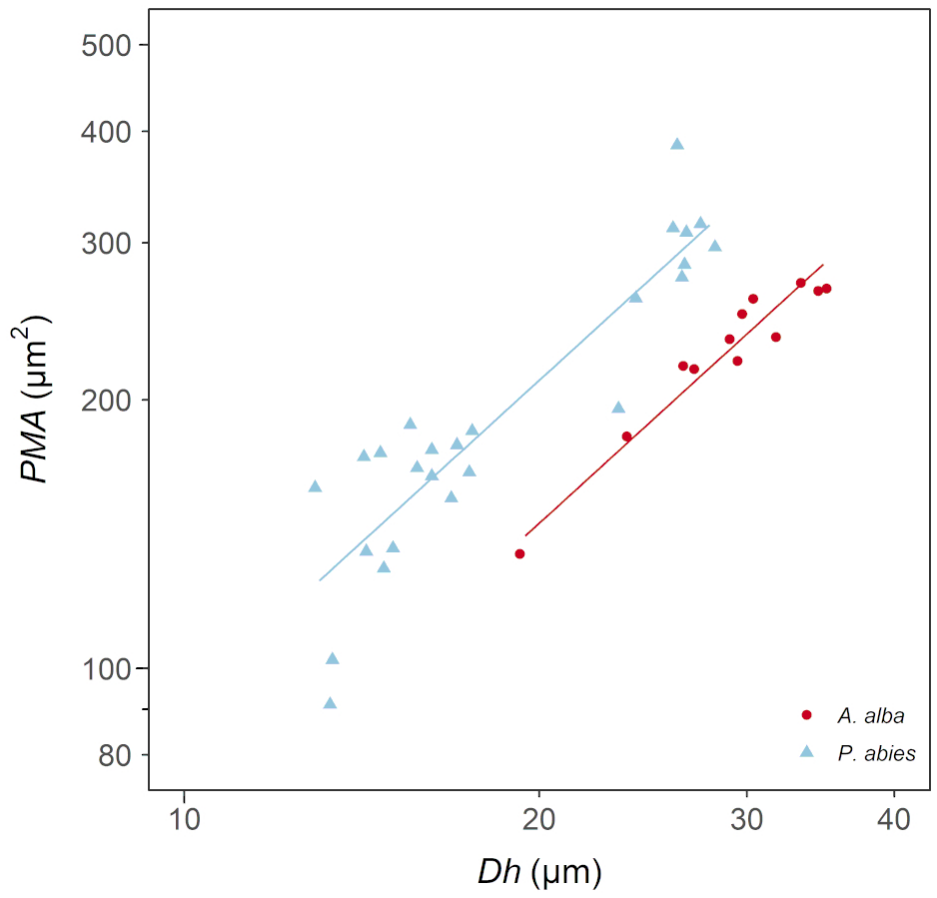
Variation in *PMA* with *Dh* in *A. alba* (blue triangles) and *P. abies* (red circles). Fitting lines are according to **Table 3**. *x* and *y* axis are shown with log_10_ scale.

We found that *PMA* increased proportionally with *Dh* according to a power relationship (*PMA*~*DFA* ^1.2^) (**Figure 4**; **Table 3**), whose exponent is coherent with the exponents characterizing the axial patterns of both *Dh* (*b*=0.19) and *PMA* (*b*=0.23) (note that 0.23/0.19 = 1.2). Furthermore, *PMA* resulted significantly larger at any observed *Dh* in the *P. abies* than the *A. alba* tree (i.e., higher *y-*intercept: **Figure 3**; **Table 3**).

**Table 3:** Results of the linear mixed-effects model with best AIC predicting the effects of *log_10_Dh* and *Tree ID* on *log_10_PMA*.

Model: *log_10_PMA* ~ *log_10_Dh + Tree ID + (Sample ID)*					
Fixed effects	Value	Std. error	DF	t value	p value
*y*-intercept	0.60	0.13	34	4.45	<0.0001
*log_10_Dh*	1.20	0.09	34	13.25	<0.0001
*Tree ID (P. abies)*	0.16	0.03	34	5.98	<0.0001
*R^2^marginal* = 0.84 *R^2^conditional* = 0.98					

*Sample ID* was used as random factor.

*P50* became progressively less negative along the stem from the apex to base, with ~1 MPa of the observed variation confined within the most apical 1.5 m of the stem (**Figure 1C**; **Figure 3C**). This *P50* axial pattern was well described with a power relationship between -*P50* and *DFA* (-*P50*~*DFA* ^-0.21^) (**Figure 3C**; **Table 2C**). -*P50* decreased at similar rates in both sampled trees (i.e., significantly similar exponent) but at any *DFA* position, the *P. abies* tree resulted more vulnerable to air seeding than the *A. alba* tree (i.e., less negative *P50*; higher *y-*intercept). Notably, the effects of *DFA* and tree *ID* explained 90% of the total *P50* variance. More specifically, the model accurately captured the P50 variation up to DFA ~800 cm. Beyond this point, the axial pattern became less clear.

**Figure 5:**
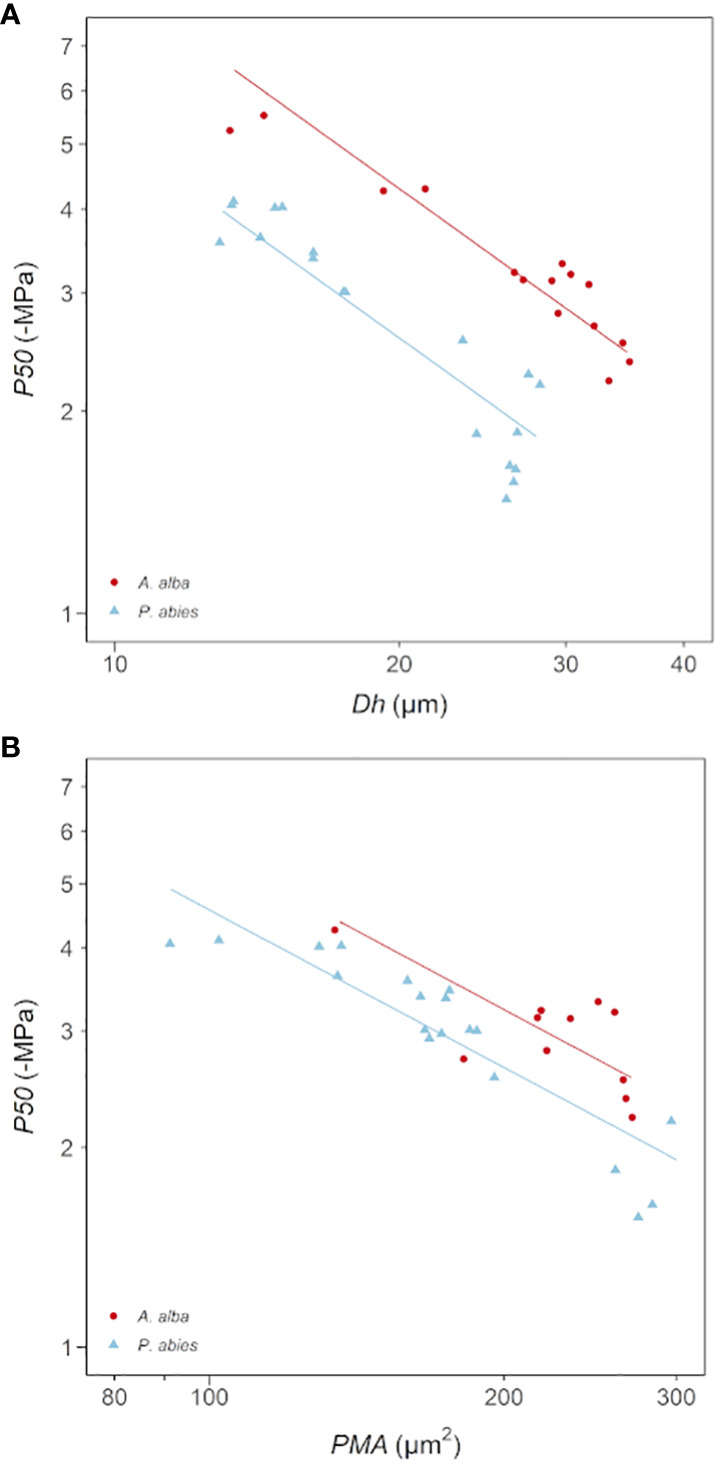
Variation in *P50* with **(A)** *Dh* and **(B)** *PMA* in *A*. *alba* (blue triangles) and *P. abies* (red circles). Fitting lines are according to **Table 4**. *x* and *y* axis are shown with log_10_ scale.

Coherently with the observed relationships between *Dh*, *PMA* and ǀ*P50*ǀ with *DFA*, we found significant power scaling relationships of -*P50* vs. *Dh* and -*P50* vs. *PMA* in both species (**Figure 5**; **Table 4**). *P50* becomes progressively more negative with increasing *Dh* and *PMA* at significantly similar rates in the two sampled trees (-*P50*~*Dh* ^-1.01^, -*P50*~*PMA* ^-0.79^). Tracheids of given *Dh* resulted more vulnerable (i.e., less negative *P50*) in the *P. abies* than the *A. alba* tree (i.e., lower *y-*intercept: **Figure 5A**; **Table 4A**). Seemingly, tracheids of given *PMA* resulted more vulnerable (i.e., less negative *P50*) in the *P. abies* than the *A. alba* tree (i.e., lower *y-*intercept: **Figure 5B**; **Table 4B**).

**Table 4:** Results of the linear mixed-effects models with best AIC predicting the effects of *Tree ID* and (A) *log_10_Dh* and (B) *log_10_PMA* on *log_10_P50*.

A	Model: *log_10_P50 ~ log_10_Dh + species + (ID)*					
	Fixed effects	Value	Std. error	DF	t value	p value
	*y*-intercept	1.95	0.11	31	18.01	<0.0001
	*log_10_Dh*	-1.01	0.08	31	-13.36	<0.0001
	*Tree ID (P. abies)*	-0.22	0.02	31	-10.31	<0.0001
	*R^2^marginal* = 0.86 *R^2^conditional* = 0.98					
**B**	Model: *log_10_P50 ~ log_10_PMA + species + (ID)*					
	Fixed effects	Value	Std. error	DF	t value	p value
	*y*-intercept	2.33	0.17	31	13.72	<0.0001
	*log_10_PMA*	-0.79	0.07	31	-11.02	<0.0001
	*Tree ID (P. abies)*	-0.09	0.02	31	-3.92	<0.0001
	*R^2^marginal =* 0.79 *R^2^conditional =* 0.97					

*Sample ID* was used as random factor.

It can be summarized that (i) the power scaling relationships of *Dh*, *PMA* and *P50* with *DFA* showed no differences in the scaling exponent between the two sampled trees, (ii) at any *DFA* position each trait differed between the sampled trees by a constant factor equal to the ratio of their allometric constant *a* (Equation 2). In practice, at any *DFA* position along the stem, tracheids of the *P. abies* tree had 20% narrower *Dh*, similar *PMA* and 35% less negative *P50*.

## Discussion

The study revealed significant axial patterns in all analyzed traits (*Dh*, *PMA* and *P50*). The increase in *Dh* and *PMA* from stem apex to base aligned with previous anatomical analyses on conifers (Anfodillo et al., 2006, 2012; Lintunen et al., 2010; Petit et al., 2011, 2023; Jyske and Hölttä, 2015; Lazzarin et al., 2016). Besides, our hydraulic measurements provided new empirical evidence of a consistent axial pattern in *P50*, becoming less negative with increasing distance from the stem apex (*DFA*). Trait variation was very clear from the stem apex down to approximately 8 m. Beyond this point, all traits showed little change.

### The functional allometry of tracheid and pit traits

All traits exhibited allometric scaling with *DFA*, following power relationships (Equation 2). These relationships are commonly found in all living organisms, representing fundamental functional properties (West et al., 1997, 1999). When one trait varies, several others change proportionally to maintain essential functional properties. The scaling exponent (*b*) describes the relative variation in the *Y* trait for a given change in the *X* trait. For example, trees maintain their upright position by increasing stem diameter (*D*) in precise proportion to the increase in tree height (*H*) to ensure mechanical stability (resulting in the universal scaling of *H*~*D* ^2/3^: McMahon & Kronauer, 1976; Niklas, 1995).

Among the traits analyzed in this study, the hydraulically weighted tracheid lumen diameter (*Dh*) received the most attention. In our trees, *Dh* increased with *DFA* ^0.2^ (scaling exponent *b*=0.2), consistent with previous studies on axial *Dh* variation along the youngest/outermost xylem ring (Anfodillo et al., 2006, 2012; Lintunen et al., 2010; Petit et al., 2011, 2023; Jyske and Hölttä, 2015). This axial pattern has been traditionally referred to as “conduit tapering” (the progressive reduction in lumen diameter from the stem base towards the apex: Schulte, 2012; Pfautsch et al., 2018). However, the axial *Dh* variation is strongly *DFA*-dependent, since such a pattern was found to be rigidly reiterated at every year of growth (i.e., the same pattern can be found along each annual ring from the ring apex to the stem base: Prendin et al., 2018; Petit et al., 2023). This pattern, where *Dh* increases with *DFA*, known as “widening”, has been hypothesized to play a crucial role in enhancing the hydraulic properties of xylem architecture. On one hand, larger conduits towards the stem base would contribute very little to the total hydraulic resistance to water transport (Becker et al., 2000; Petit and Anfodillo, 2009), thus effectively contributing to maintain an efficient leaf water supply while growing taller (West et al., 1999; Petit et al., 2010). On the other hand, increasingly conductive conduits from the stem apex to base would strongly affect the shape of the water potential (*Ψ*) gradient from the stem apex to base, with the lowest (i.e., most negative) *Ψ* concentrated within a short distance from the apex (Lechthaler et al., 2020).

Notably, the above conditions received indirect empirical support from studies reporting very limited variation in the contribution of pit resistance to the total conduit (either vessels in angiosperms and tracheids in conifers) resistance with increasing conduit diameter (Domec et al., 2006; Pittermann et al., 2006; Petit et al., 2008).

### Tracheid-pit allometry

Tracheid diameter and different pit traits have been reported to be highly correlated both at the individual (Schulte, 2012; Lazzarin et al., 2016; Held et al., 2021) and interspecific level (Hacke et al., 2004; Delzon et al., 2010).

Our measurements of *PMA* and *Dh* at different positions along the stem provided new evidence that tracheid and pit traits tightly vary in tandem along the stem from apex to base (Schulte, 2012; Lazzarin et al., 2016). Both *PMA* and *Dh* varied with *DFA^b^*, with the scaling exponent for both relationships and in both *A. alba* and *P. abies* tree being *b*~0.2, thus very similar to the same scaling relationships reported for the giant *Sequoiadendron giganteum* (Lindl.) J.Buchh. tree analyzed by Lazzarin et al. (2016).

Furthermore, Lazzarin et al. (2016) reported highly significant *DFA*-dependent trends not only for *PMA*, but also for torus area and pit aperture area. Since these traits are typically highly correlated (Hacke et al., 2004; Delzon et al., 2010), it seems highly likely that also in our analyzed trees the torus area and pit aperture area conformed to these relationships with *PMA*, and therefore progressively increased with *DFA* (additional indirect evidence is discussed below).

The regulation of the tracheid lumen diameter, as well as pit traits, is likely associated to the duration of cell elongation, which progressively increases from the stem apex to base (Anfodillo et al., 2012).

### Increasing embolism vulnerability from the stem apex to base

While it is widely acknowledged that pit traits (*PMA*, torus area and pit aperture area) strongly influence the resistance against embolism formation through air seeding via pit membranes (Hacke et al., 2004; Delzon et al., 2010; Bouche et al., 2014), the growing body of empirical evidence demonstrating strong and consistent (almost universal) anatomical patterns dependent on *DFA* has not adequately motivated the scientific community to investigate the impact of *DFA* on xylem hydraulic properties, such as embolism vulnerability.

We observed a tremendous variation in *P50* (>3 MPa) along the stem of both the *P. abies* and *A. alba* trees. This axial variability is twice as large as that reported for *Pseudotsuga menziesii* using the same air-injection technique (Domec and Gartner, 2001). In contrast, Bouche et al. (2016) reported no differences in *P50* of the outer sapwood at different heights along the trunk of mature conifer individuals. Notably, our data are consistent with both studies because neither of them measured samples extracted at a short distance from the apex. More specifically, Domec & Gartner did not sample segments between the stem apex and the fifth internode, likely located at a distance of over 1 meter from the stem apex, whereas Bouche et al. cut the most apical trunk bole segment with a chainsaw, therefore likely at an even greater distance from the apex. Seemingly to Bouche et al. (2016), the *P50* variation along the stem of our trees from ~3 m from the apex until the stem base was very limited and hardly significant if extrapolated from the remaining distal part of the stem. Furthermore, the more negative *P50* of 1-cm-diameter branches compared to that of trunk in Bouche et al. (2016) is also consistent with our results.

Our *P50* data from segments at short distance from the apex of both trees were in line with those found in the literature and obtained from *VCs* of apical branches (<1 m) measured with different techniques (bench dehydration and hydraulic measurements, acoustic, centrifugation, etc.) (Choat et al., 2012; Feng et al., 2021).

Even if sometimes criticised, especially when applied to long- and wide-vesseled species (Laughlin et al., 2023), the air injection method is well established for analyses on conifers and short-vesseled species, and several studies showed good agreements between the air injection and other methodical approaches (Rosner et al., 2019a).

The axial pattern of *P50* strongly depended on *DFA*, following a power trajectory similar to *Dh* and *PMA* (ǀ*b*ǀ~0.2). However, it also resembled the trajectory of torus area and pit aperture area (Lazzarin et al., 2016). *P. abies* exhibited less negative *P50* (**Figure 3C**; **Table 2C**), narrower *Dh* (**Figure 3A**; **Table 2A**) and similar *PMA* (**Figure 3B**; **Table 2B**) compared to *A. alba* along the stem. It would be a mistake to interpret this as the *P. abies* tree being more vulnerable due to its narrower tracheids. Instead, this comparison simply showed that vulnerability is not determined by tracheid diameter alone (Lens et al., 2022), but rather by other traits that are correlated with it. Tracheids with a given *PMA* showed higher vulnerability to gas-embolism in the *P. abies* than in the *A. alba* tree (lower *y*-intercept: **Table 4B**; **Figure 5B**). However, both trees exhibited similar *PMA* at any *DFA* position (**Table 2B**; **Figure 3B**), while *P50* differed (**Table 2C**; **Figure 3C**). This could potentially be explained by other pit traits, such as torus area and the aperture area, that typically vary proportionally with *Dh* and *PMA* (Lazzarin et al., 2016). Most likely, at any *DFA* and *PMA*, the *P. abies* tree resulted more vulnerable than the *A. alba* tree due to its narrower torus area and/or larger pit aperture area, corresponding to a less efficient torus overlap (Hacke and Jansen, 2009). Indeed, the correlation between different anatomical traits (tracheid diameter, pit membrane area, torus area and aperture area) is typically very high (Delzon et al., 2010; Bouche et al., 2014; Held et al., 2021), in agreement with our data and the findings on axial trends in anatomical traits (Schulte, 2012; Lazzarin et al., 2016).

The within-tree *P50* variability reported in this study for only two trees corresponded to approximately 60% of the *P50* variability among many gymnosperm species reported in a previous study (Choat et al., 2012).

To ensure comparability of xylem hydraulic analyses, it is crucial to consider the potential *DFA* effects on lumen diameter, pit traits, and hydraulic characteristics.

Xylem hydraulic data, such as *P50* and xylem specific conductivity (*ks*), are commonly obtained by sampling stem/branch segments with standardized age (i.e., number of rings) or diameter, and specific length (depending on methods), thus they do not account for *DFA* effects. Therefore, if *DFA* is unknown, accurate comparability of published data is quite challenging.

### Towards the allometric scaling of plant hydraulics

The observed increase in *P50*, becoming less negative, along the stem from apex to base is in line with the predicted pattern based on the axial variation in tracheid and pit traits mentioned earlier.

Removing the *DFA* effect from hydraulic measurements is not a straightforward task. In our trees -*P50* scaled with *DFA* ^-0.2^. However, our limited data cannot exclude that *b* may be higher or lower than -0.2 depending on species and/or environmental conditions. Therefore, sampling at fixed *DFA* does not entirely eliminate the *DFA* effects on *P50*, unless the *b*=-0.2 will be demonstrated to be universal in plants.

**Figure 6:**
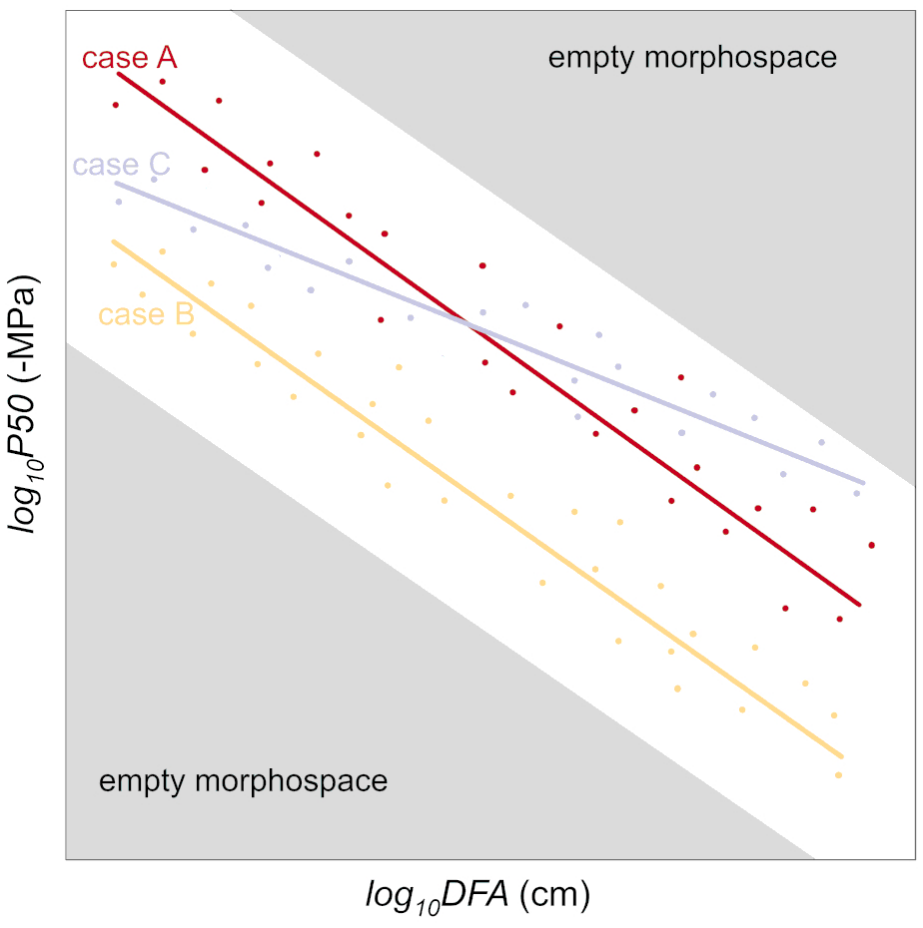
How allometric trajectories can be used for studying the variability of *P50* (or other hydraulic traits) in different contexts. The base case A represents the *P50* variation with *DFA* at the single individual level. Compared to case A, the lower *y-*intercept of case B indicate a lower resistance against drought-induced embolism formation; the lower *y-*intercept and higher slope of case C indicate a lower embolism resistance in the upper stem and a higher resistance in the lower stem. Convergence towards a similar scaling of *P50* with *DFA* could be the result of selection eliminating variants with too high *P50* towards the apex (where the xylem tensions are the highest due to the proximity to the transpiring leaves), and too low tracheid conductivity towards the base (determining hydraulic limitations to leaf water supply).

Instead, considering the axial variability in anatomical (e.g., conduit diameter and pit traits) and hydraulic (e.g., xylem specific conductivity, *P50*) traits would help account for the *DFA* effects. Applying our allometric approach, we could determine that the analyzed *P. abies* tree was 35% more vulnerable than the *A. alba* tree: since -*P50*~*DFA^-0.2^* (i.e., similar exponent *b*=0.2), at any *DFA* position along the stem the ratio of *P50* between the two trees was equal to the ratio of their allometric constant *a* (Equation 1) (*a_P.abies_/a_A.alba_* =1.35). This relationship can be visually represented as parallel lines on a log-log graph (using either log-transformed data or a linear scale with logarithmic axes) (cf. case A vs. case B in **Figure 6**: same slope and different *y*-intercept).

In cases where two individuals would have different values for *a* and *b* (Equation 2), resulting in distinct *y*-intercepts and slopes on log-log representation (Equation 2) (cases A and C of **Figure 6**), measuring *P50* at a single position in the branch would lead to one individual (case A) appearing less vulnerable if samples were taken towards the apex, or more vulnerable if samples were taken towards the base.

Further investigations are needed to test the consistency and generality of the axial scaling of embolism vulnerability (*P50*) with *DFA*. This approach could provide a new perspective on studying plant functional traits, including anatomical and hydraulic characteristics. Empirical evidence increasingly supports the notion of species converging towards a universal scaling of conduit diameter (*Dh*) with *DFA* (Anfodillo et al., 2013; Olson et al., 2018; Kiorapostolou and Petit, 2019; Lechthaler et al., 2019; Petit et al., 2023). Additionally, there is growing evidence of similar scaling relationships between pit traits and *DFA* (this study, Schulte, 2012; Lazzarin et al., 2016). It is reasonable to assume that evolution has favored variants that strike a balance between safety and efficiency, eliminating those with vulnerable conduits towards the apex (where xylem pressures are most negative due to proximity to transpiring leaves) and those with low conductive vascular elements towards the base (which would limit leaf water supply and gas exchange efficiency). This can be visualized as empty morphospaces in **Figure 6**.

In this context, extending this common evolutionary pattern to angiosperms appears more complex due to practical challenges in testing axial variation in xylem embolism formation in broadleaved species compared to conifers. Additionally, although patterns of vessels/tracheid and pit anatomical traits are similar across studies (Petit et al., 2010; Lechthaler et al., 2019; Soriano et al., 2020), hydraulic tests carried out with different techniques (flow centrifuge, optical visualization and pneumatic air discharge) seemed not to support the hypothesis that large axial *P50* variation applies also to vessel-bearing species (Creek et al., 2018; Rodriguez-Dominguez et al., 2018; Wu et al., 2020; Lübbe et al., 2022). But notably, no studies have directly investigated the variation in *P50* with the distance from the apex in angiosperms.

## Conclusion

This study presents novel empirical evidence that supports the concept of xylem tracheids having all xylem anatomical traits strongly influenced by their distance from the apex. This results in significant variation in *P50* along the stem, from the apex to the base.

We strongly emphasize the importance for researchers investigating xylem hydraulics to carefully consider the potential impact of axial distance from the apex on traits such as embolism vulnerability. Accounting for this effect is crucial for obtaining accurate measurements, enabling robust analysis and comparison of individuals, populations, and species. Ultimately, this knowledge will enhance our ability to predict the impacts of climate change on plant communities.

## Data availability statement

The raw data supporting the conclusions of this article will be made available by the authors, without undue reservation.

## Author contributions

DZ: Data curation, Formal analysis, Investigation, Writing – review & editing. TS: Investigation, Methodology, Supervision, Writing – review & editing. SR: Conceptualization, Methodology, Writing – review & editing. GP: Conceptualization, Supervision, Visualization, Writing – original draft, Writing – review & editing.
